# Association of nitric oxide synthase gene polymorphism with asthma: A systematic review and meta‐analysis

**DOI:** 10.1111/crj.13617

**Published:** 2023-04-19

**Authors:** Zeru Fan, Tao Liu, Wei Na

**Affiliations:** ^1^ Department of Medical Insurance Heilongjiang Provincial Hospital Harbin Heilongjiang China; ^2^ Department of Respiratory Medicine Heilongjiang Provincial Hospital Harbin Heilongjiang China

**Keywords:** asthma, gene polymorphism, nitric oxide synthase, NOS, variants

## Abstract

**Introduction:**

This study examines the associations between asthma and nitric oxide (NO) synthase (NOS) gene polymorphisms.

**Methods:**

After a systematic literature search in electronic databases, studies were selected based on eligibility criteria. Data were extracted from research articles and were synthesized and tabulated. Where a particular polymorphism data were reported by multiple studies, meta‐analyses of odds ratios were performed, or odds ratios reported by individual studies were pooled.

**Results:**

Twenty studies (4450 asthma patients and 5306 non‐asthmatic individuals) were identified. Many studies did not find any association between CCTTT repeat polymorphism in NOS2 gene and asthma. However, a study reported that pretreatment mean exhaled NO levels in asthmatics were found to be significantly higher in genotypes with higher number of CCTTT repeats. Also, alleles with <11 CCTTT repeats were associated with poor asthma treatment outcomes. A single nucleotide polymorphism, G894T, in NOS3 gene was not found to be significantly associated with asthma by at least four studies. However, a T allele at this locus was associated with lower NO levels. Also, G894T frequency was significantly higher in asthmatic children who responded to inhaled corticosteroids along with long‐lasting beta2‐agonists. A T allele of NOS3 786C/T polymorphism increased the probability of bronchial asthma with comorbid essential hypertension in asthma patients. Asthma severity also differed for different Ser608Leu exon 16 variants of NOS2 gene.

**Conclusions:**

Several polymorph NOS gene variants are identified, some of which appear to have influence on asthma prevalence or outcomes. However, data are varying depending on the nature of variant, ethnicity, study design, and disease parameters.

## Graphical abstract

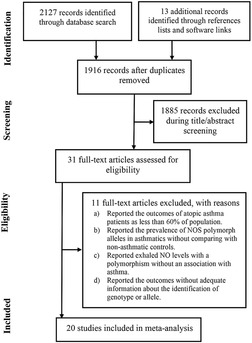

## 1 INTRODUCTION

Asthma is a multifactorial chronic inflammatory disease affecting both children and adults. It is the most common chronic disease in children. Asthma is characterized by bronchial constriction and hyperreactivity, airflow limitation, and mucous hypersecretion, which leads to symptoms such as wheezing, coughing, and shortness of breath.^1^ Increase in secretions, reduced airway pliability, and the presence of mucosal edema may further increase the bronchoconstriction.^2^ Structural and functional alterations in respiratory epithelium play a crucial role in asthma pathophysiology. Such alterations include airway wall thickening, subepithelial fibrosis, myocyte hypertrophy, myofibroblast hyperplasia, and mucus metaplasia.^3^

The prevalence of asthma is considerably high. In USA, the prevalence of asthma is 12.8% (https://www.cdc.gov/asthma/nhis/2019/data.htm); whereas in China, its prevalence is estimated at 4.3%, which can be underestimated because of underdiagnosis.^4^ In 2019, 262 million people suffered from asthma worldwide. Mortality due to asthma is higher in low‐income countries where asthma remains underdiagnosed and undertreated (https://www.cdc.gov/asthma/nhis/2019/data.htm; https://www.who.int/news-room/fact-sheets/detail/asthma). Cigarette smoking, allergic rhinitis, childhood pneumonia or bronchitis, parental history of a respiratory disease, and low educational attainment are identified as risk factors for adult asthma.^4^ Obesity is also an important risk factor for the development of asthma (https://www.cdc.gov/asthma/nhis/2019/data.htm).

Nitric oxide (NO) is a ubiquitous signaling molecule acting as an important vasodilator, neurotransmitter, and inflammatory mediator. However, it also plays a role in the pathophysiology of asthma. NO is synthesized by the NO synthase (NOS) enzymes, which are found to be of three types. NOS1 is mainly expressed by the noradrenergic, non‐cholinergic nerve fibers of the airway. NOS2 is mainly found in inflammatory immune cells and is also present in the respiratory epithelium. NOS3 is mainly found in the endothelial cells but it is also observed in the bronchiolar and alveolar cells.^5^ NO is formed by the NOS enzymes when L‐arginine is converted to L‐citrulline. NO is a potent molecule for the recruitment of inflammatory cells and for the amplification of inflammatory response.^6^

One of the major anatomical locations where NO is produced is the paranasal sinuses where it is synthesized by the NOA2A. Whereas NO is constitutively expressed in nasal sinus epithelial surfaces, it is not generally expressed in the nasal cavity.^7^ Exhaled air of the asthmatic patients contains higher levels of NO that can be reduced upon corticosteroid treatment.^6^ Exhaled NO acts as an inflammatory biomarker of the airway^8^ and high levels of exhaled NO can be used to classify asthma severity and helps in identifying patients at risk.^9^

Asthma predisposition involves gene–environment interactions in its onset as well as severity. Etiology of asthma has genetic elements, and its heritability is reported to be between 36% and 77%. Over 100 genes are implicated for the pathogenesis of asthma and related conditions.^10^ Polymorphisms are observed in several genes related to asthma pathophysiology, and NOS genes also exhibit multiple variants. The objective of the present study was to conduct a systematic literature search for the identification of the studies that reported the association between a NOS gene variant and asthma prevalence or severity.

## 2 MATERIALS AND METHODS

### 2.1 Inclusion and exclusion criteria

The studies included in this systematic review are those that (a) examined the association between NOS gene polymorphism and asthma incidence or severity; (b) reported statistical indices of the relationship between a gene variant and incidence or severity of asthma; (c) reported incidence of asthma in a cohort of individuals with a particular NOS variant; and (d) reported the association between NOS polymorphism and IgE levels in asthma patients. Studies were, however, excluded if they (a) reported the outcomes of atopic asthma patients but their proportion was less than 60% in the study population; (b) reported the prevalence of NOS polymorph alleles in asthmatics without comparing with non‐asthmatic controls; and (c) reported the outcomes without adequate information about the identification of genotype or allele.

### 2.2 Literature search

The literature search was conducted in electronic databases (Google Scholar, Ovid, PubMed, Science Direct, and Wiley). Most relevant keywords were used for literature search by using these as phrases: asthma, nitric oxide synthase, NOS, NOS1, NOS2, NOS3, inducible, iNOS, endothelial, eNOS, gene polymorphism, variants, genetic variations, repeat, copy, promoter, intron, and phenotype. The literature search encompassed original research articles published from the data of database inception until October 2021. After the identification of relevant research articles, the references lists of these articles were also screened for additional records.

### 2.3 Data synthesis and analyses

Demographic, allelic or genotypic, polymorph or variant data, prevalence in asthmatics and non‐asthmatics, associational statistics including odds ratios and regression coefficients, and immunoglobin E (IgE) levels in asthmatics with a particular allele variant were extracted from the research articles of respective studies and synthesized according to the type of NOS or the presence of polymorphism. For studies that reported the descriptive data regarding the prevalence of polymorphism in association with asthma or its severity, odds ratios were calculated. For polymorphic genes whose outcomes were reported by more than one study, the odds ratios were pooled using DerSimonian–Liard method to achieve overall estimates.

## 3 RESULTS

**FIGURE 1:**
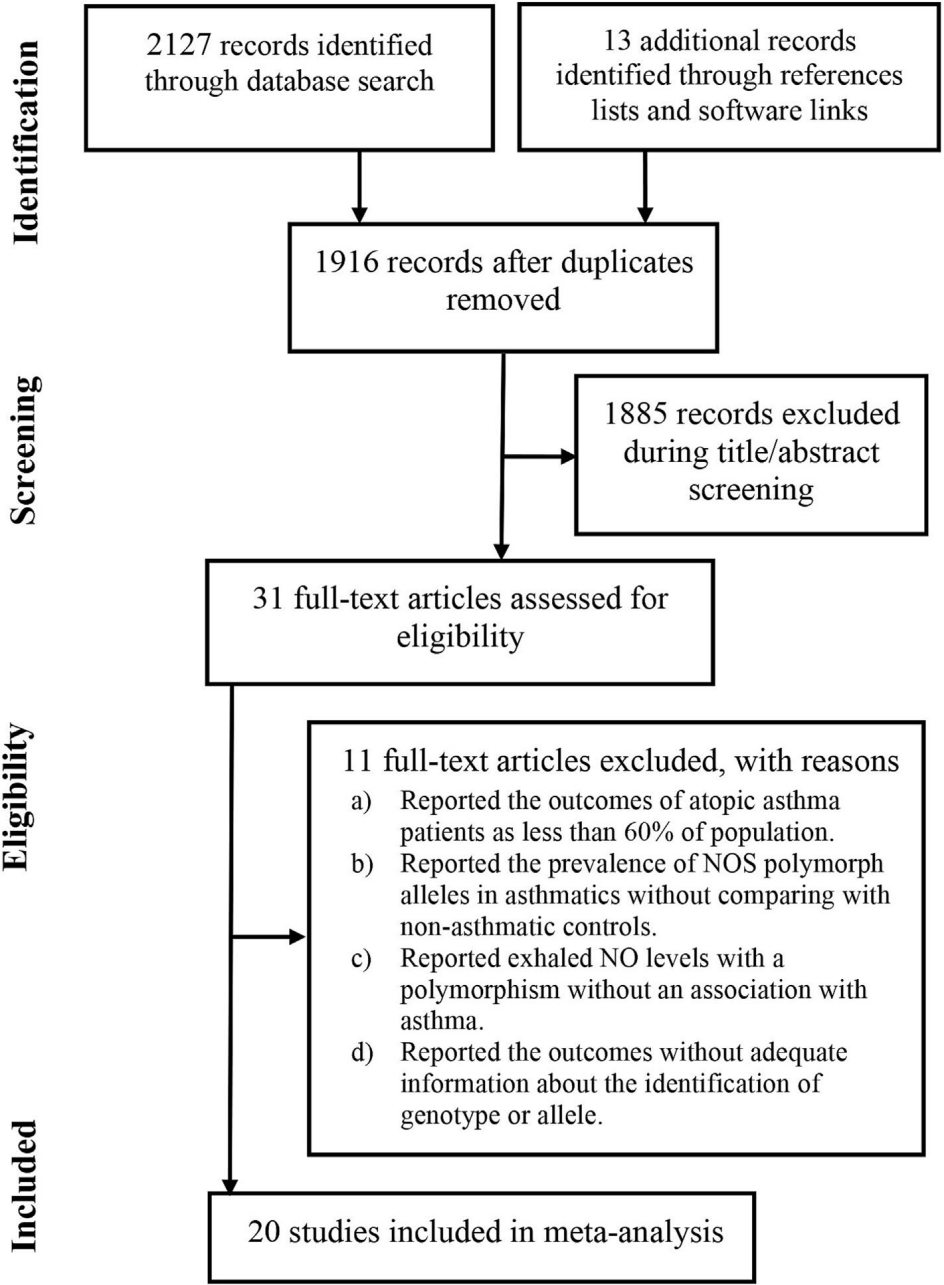
A flowchart of study screening and selection process.

Twenty studies were identified from the literature by following the eligibility criteria.^11^, ^12^, ^13^, ^14^, ^15^, ^16^, ^17^, ^18^, ^19^, ^20^, ^21^, ^22^, ^23^, ^24^, ^25^, ^26^, ^27^, ^28^, ^29^, ^30^ A flowchart of study screening and selection process is presented in Figure 1. In these studies, 4450 asthmatic patients and 5306 non‐asthmatics were recruited and studied for NOS polymorphism. The age of asthma patients were 29.9 years [95% confidence interval (CI): 23.2, 36.5] (range 8.5 ± 0.2 to 60 ± 1.1) and 47% [95% CI: 40, 53] of these patients were females.

Several authors have reported the odds ratios depicting an association between a NOS gene polymorphism and asthma risk. Table 1 shows NOS gene polymorphisms, many of which had a statistically significant association with asthma. Several studies did not report the odds ratios of the association between a polymorphic gene and asthma risk but provided numeric data showing the prevalence of asthma in carriers and noncarriers. For these studies, odds ratios were calculated from raw data and are presented in Table 2.

**TABLE 1:** Odds ratios of the association of NOS polymorphism with asthma.

Study	Polymorph allele	Association	OR [95% CI]; *p*‐value
NOS1			
Gao 2000	Homozygous 183‐bp alleles with VNTR in intron 2	Higher risk of asthma vs controls in carriers	2.08 [1.2, 3.57]; *p* = 0.01
Grasemann 2000	Allele with 17 CA‐repeat in exon 29	Higher risk of asthma vs controls in carriers	1.49 [1.17, 1.9]; *p* = 0.0013
Grasemann 2000	Allele with 18 CA repeat in exon 29	Lower risk of asthma vs controls in carriers	0.49 [0.3, 0.8]; *p* = 0.0006
Grasemann 2000	Allele with 17 CA repeat in exon 29	Risk of high IgE levels in asthmatic carriers	8.2 [5.93, 11.3]; *p* < 0.0001
Grasemann 2000	Allele with 18 CA repeat in exon 29	Risk of high IgE levels in asthmatic carriers	8.15 [5.9, 11.3]; *p* < 0.0001
Holla 2004	SNP with C/T transition located 276‐bp downstream of translation termination site (C5266T) in exon 29	Higher risk of serum IgE levels in T allele carriers	2.08 [1.52, 2.85]; *p* < 0.01
Wechsler 2000	Alleles with >12 AAT repeats	Lower risk of asthma in carriers	0.62 [0.43, 0.89]; *p* = 0.01
NOS2			
Gao 2000	Homozygous Glu298Asp	No significant risk of asthma	0.73 [0.46, 1.17]; *p* = 0.19
Batra 2007	Allele with 3 GT repeats in intron 4	Higher risk of severe asthma in carriers	2.62 [1.03, 6.6]; *p* = 0.04
Hirai 2018	Alleles with <11 CCTTT repeats	Higher risk of asthma exacerbations in carriers	2.8 [1.2, 6.6]; *p* = 0.016
Holla 2006	T allele with Ser608Leu polymorphism in exon 1	Higher risk of asthma severity in carriers	5 [1.88, 13.3]; *p* = 0.0005
NOS3			
Gao 2000	Homozygous 311 bp allele with 5′ promoter VNTR	No significant risk of asthma	1.07 [0.6, 1.92]; *p* = 0.81
Bouzigon 2012	rs743507 (Major allele T and minor allele C)	Higher risk of high FeNO in asthmatics	Beta coefficient 0.08 [0.03, 0.14]; *p* = 0.004
Holla 2008	‐786rs2070744	Higher risk of asthma in CC vs TT genotype	1.6 [0.97, 2.66]; *p* = 0.028
Holla 2008	‐786rs2070744	Higher risk of asthma CT vs TT genotype	1.27 [0.9, 1.8]; *p* = 0.028
Holla 2008	‐691rs3918226	Higher risk of asthma CT vs CC genotype	1.67 1.05, 2.64]; *p* = 0.039
Holla 2008	G11Trs1799985	Lower risk of asthma GT vs GG genotype	0.81 [0.57, 1.14]; *p* = 0.039
		Lower risk of asthma TT vs GG genotype	0.67 [0.42, 1.1]; *p* = 0.043
Holla 2008	27‐bp repeat	No significant risk of asthma aa vs bb genotypes	1.2 [0.52, 2.78]
		No significant risk of asthma ab vs bb genotypes	1.05 [0.74, 1.49]
Holla 2008	774rs1549758	No significant risk of asthma CT vs CC genotypes	1.13 [0.81, 1.57]
		No significant risk of asthma TT vs CC genotypes	1.09 [0.57. 2.06]
Holla 2008	894rs1799983	No significant risk of asthma GT vs GG genotypes	1.18 [0.85, 1.65]
		No significant risk of asthma TT vs GG genotypes	1.22 [0.68, 2.19]
Shakhanov 2017	786C/T	Higher risk of comorbid asthma and essential hypertension compared with bronchial asthma alone	2.40 [1.04, 5.56]
van's Gravesande 2003	G894T	No significant risk of asthma	1.07 [0.51, 2.25]; *p* = 0.86
van's Gravesande 2003	G894T	No significant risk of asthma	2.50 [0.61, 10.3]; *p* = 0.2

Abbreviations: IgE, immunoglobin E; NOS, nitric oxide synthase, OR, odds ratio.

**TABLE 2:** Association of NOS polymorphism with asthma.

Study	Polymorphic allele	Genotype	Asthmatics		Non‐asthmatics		Odds ratio [95% CI]; *p*‐value
			Carriers	Noncarriers	Carriers	Noncarriers	
NOS1							
Gao 2000	183‐bp allele with VNTR in intron 2	Homozygous 183‐bp	62	24	119	95	2.06 [1.2, 3.55]; ***p* = 0.009**
Holla 2004	C/T polymorph in exon 29	CC	83	174	67	146	1.04 [0.7, 1.54]; *p* = 0.846
		CT	56	120	94	200	0.99 [0.66, 1.48]; *p* = 0.972
		TT	11	26	139	294	0.89 [0.43, 1.86]; *p* = 0.767
Leung 2005	AAT‐repeat	>12/>12 repeats	49	42	52	58	1.3 [0.75, 2.27]; *p* = 0.354
		Heterozygous	15	20	86	80	0.7 [0.33, 1.46]; *p* = 0.337
		<12/<12 repeats	37	38	64	62	0.94 [0.53, 1.67]; *p* = 0.841
Leung 2005	C5266T SNP	CC	45	42	56	58	1.11 [0.63, 1.94]; *p* = 0.715
		CT	44	44	57	56	0.98 [0.56, 1.72]; *p* = 0.95
		TT	11	14	90	86	0.75 [0.32, 1.74]; *p* = 0.505
NOS2							
Gao 2000	311 bp allele with 5′ promoter VNTR	Homozygous 311‐bp	144	96	37	23	0.93 [0.52, 1.67]; *p* = 0.813
Konno 2001	CCTTT‐repeat	Non‐14/non‐14 repeat	229	25	220	23	0.96 [0.53, 1.74]; *p* = 0.887
		Heterozygous	22	232	22	221	0.95 [0.51, 1.77]; *p* = 0.878
		14/14 repeat	3	251	1	242	2.89 [0.30, 28.0]; *p* = 0.359
Leung 2006	CCTTT‐repeat	Non‐14/non‐14 repeat	241	143	50	29	0.98 [0.59, 1.61]; *p* = 0.929
		Heterozygous	49	27	242	145	1.09 [0.65, 1.82]; *p* = 0.749
		14/14 repeat	1	2	290	170	0.29 [0.03, 3.26]; *p* = 0.318
NOS3							
Gao 2000	Glu298Asp	Homozygous Glu	96	54	85	65	1.36 [0.85, 2.16]; *p* = 0.195
Holla 2002	A549G	AA	87	111	39	41	0.82 [0.49, 1.39]; *p* = 0.466
		AG	35	39	91	113	1.11 [0.65, 1.90]; *p* = 0.691
		GG	4	2	122	150	2.46 [0.44, 13.7]; *p* = 0.304
Holla 2008	‐691rs3918226	CC	241	278	53	38	0.62 [0.4, 0.98]; *p* = 0.039
		CT	52	36	242	280	1.67 [1.06, 2.64]; *p* = 0.028
		TT	1	2	293	314	0.54 [0.05, 5.94]; *p* = 0.611
Holla 2008	774rs1549758	CC	145	165	149	151	0.89 [0.65, 1.22]; *p* = 0.475
		CT	128	129	166	187	1.12 [0.81, 1.54]; *p* = 0.498
		TT	21	22	273	294	1.03 [0.55, 1.91]; *p* = 0.931
Holla 2008	G11Trs1799985	GG	131	121	163	195	1.30 [0.94, 1.79]; *p* = 0.117
		GT	125	143	169	173	0.89 [0.65, 1.23]; *p* = 0.496
		TT	38	52	256	264	0.75 [0.48, 1.18]; *p* = 0.22
Lee 2000	Endothelial constitutive NOS	bb	91	272	30	38	0.42 [0.25, 0.72]; ***p* = 0.002**
		ab	29	35	92	275	2.48 [1.43, 4.27]; ***p* = 0.001**
		aa	1	3	120	307	0.85 [0.09, 8.28]; *p* = 0.891
Yanamandra 2005	VNTR in intron 4	aa	7	18	107	318	1.16 [0.47, 2.84]; *p* = 0.753
		bb	60	196	54	140	0.79 [0.52, 1.22]; *p* = 0.289
		cc	0	0	114	336	
		ab	36	103	78	233	1.04 [0.66, 1.65]; *p* = 0.854
		ac	7	5	107	331	4.33 [1.35, 13.9]; ***p* = 0.014**
		bc	6	14	108	322	1.28 [0.48, 3.41]; *p* = 0.624

Abbreviation: NOS, nitric oxide synthase.

**FIGURE 2:**
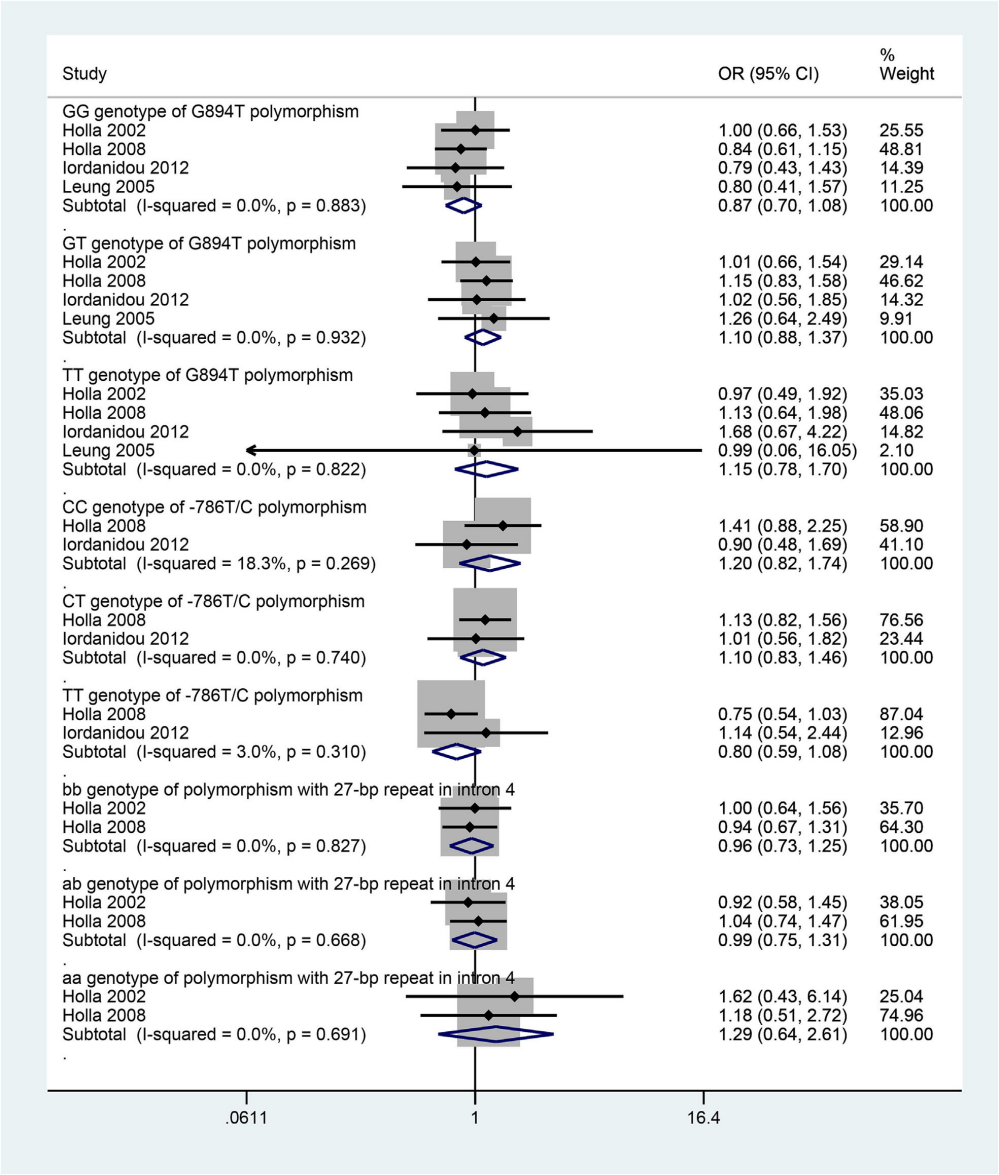
Forest plots showing the pooled odds ratios of the associations between asthma and three genotypes of G894T variant of NOS3 gene (Glu298Asp); three genotypes of a polymorphism of NOS3, −786 T/C; and three genotypes of a 27 bp repeat polymorphism of NOS3.

A very few gene polymorphisms were reported by more than one individual study. A meta‐analysis of four studies found that G894T variant of NOS3 gene (Glu298Asp) was not found to be significantly associated with asthma risk (Figure 2). A meta‐analysis of two studies found that a polymorphism of NOS3, −786 T/C, was also not significantly associated with asthma risk (Figure 2). Another meta‐analysis of two studies found that a 27‐bp repeat polymorphism of NOS3 was also not significantly associated with asthma risk (Figure 2).

Table 3 presents the outcomes of the studies that reported the association between NOS gene polymorphism and IgE levels in asthma patients. In asthmatics, a polymorphism in exon 29 of NOS1 gene (homozygous for C allele) was associated with lower IgE levels. A homozygous genotype for <12 AAT repeat was found to be associated with significantly lower IgE levels; whereas, a heterozygous genotype (<12 and >12 AAT repeat) was significantly associated with higher IgE levels in asthmatic patients.

**TABLE 3:** Association between NOS polymorphism and IgE levels in asthma patients.

Study	Polymorphic allele	Genotype	High IgE levels		Low IgE levels		Odds ratio [95% CI]; *p*‐value
			Carriers	Noncarriers	Carriers	Noncarriers	
NOS1							
Gao 2000	183‐bp allele with VNTR in intron 2	Homozygous 183‐bp	45	88	41	126	1.572 [0.95, 2.60]; *p* = 0.078
Holla 2004	C/T polymorphism in exon 29	CC	71	78	129	87	0.614 [0.40, 0.94]; *p* = **0.023**
		CT	61	88	73	143	1.358 [0.88, 2.09]; *p* = 0.165
		TT	17	132	14	202	1.858 [0.89, 3.90]; *p* = 0.101
Leung 2005	C5266T	CC	124	171	80	94	0.852 [0.58, 1.24]; *p* = 0.406
		CT	127	168	78	96	0.930 [0.64, 1.36]; *p* = 0.708
		TT	41	254	16	158	1.594 [0.87, 2.94]; *p* = 0.135
Leung 2005	AAT‐repeats	<12/<12 repeats	41	254	38	136	0.578 [0.36, 0.94]; *p* = **0.028**
		Heterozygous	148	147	68	106	1.569 [1.07, 2.30]; *p* = **0.02**
		>12/>12 repeats	109	186	70	104	0.871 [0.59, 1.28]; *p* = 0.48
NOS2							
Gao 2000	311 bp allele with 5′ promoter VNTR	Homozygous 311‐bp	105	28	135	32	0.889 [0.50, 1.57]; *p* = 0.684
Leung 2006	CCTTT‐repeat	non‐14/non‐14 repeat	231	60	148	24	0.624 [0.37, 1.05]; *p* = 0.074
		Heterozygous	45	246	31	141	0.832 [0.50, 1.38]; *p* = 0.473
		14/14 repeat	1	290	2	170	0.293 [0.03, 3.26]; *p* = 0.318
NOS3							
Gao 2000	Glu298Asp	Homozygous Glu	67	66	83	84	1.027 [0.65, 1.62]; *p* = 0.907
Leung 2005	G894T	GG	227	68	138	36	0.871 [0.55, 1.37]; *p* = 0.552
		GT	65	230	35	139	1.122 [0.71, 1.78]; *p* = 0.624
		TT	3	292	2	172	0.884 [0.15, 5.34]; *p* = 0.893

Abbreviations: IgE, immunoglobin E; NOS, nitric oxide synthase, VNTR, variable number of tandem repeats.

## 4 DISCUSSION

In this systematic review, we have identified 21 studies that evaluated the association between asthma and one or more polymorphisms in a NOS gene. Several NOS gene polymorphisms are found to have significant association with asthma. Moreover, a few polymorphisms were found to have significant associations with IgE levels in asthma patients. However, many of the polymorphisms were reported only by a single study.

A number of studies have evaluated the role of a polymorphism in NOS2 gene characterized by the CCTTT repeats. Konno et al (2001) described 14‐repeat CCTTT allele as a potentially susceptible or disease modifying allele of inflammatory immune diseases such as atopy. They found significantly higher prevalence of the 14‐repeat CCTTT allele in the NOS2 promoter in non‐atopic individuals (odds ratio [OR] for the presence of atopy between carriers and non‐carriers was 0.42 [95%CI: 0.23, 0.79]). The OR for the development of atopy was independent of asthma, as the genetic effect of the 14‐repeat CCTTT allele was persistent even after controlling the age, sex, smoking, and asthma status. Atopic asthma patients constituted 66% of this population.^22^ Leung et al (2006) also found no association between CCTTT repeats and asthma or exhaled NO in Chinese asthma patients.^25^

Batra et al (2007) did not find any significant association between 14‐repeat CCTTT allele with asthma in Indian asthma patients.^11^ Pascual et al (2008) also did not find any association between CCTTT repeats in the NOS2A and atopic asthma in a Spanish population, in which increasing number of CCTTT repeat was associated with nasal polyposis instead.^26^ However, Sato et al (2016) found that the mean exhaled NO levels before treatment in Japanese asthmatic patients were significantly higher in genotypes with higher number of CCTTT repeats (ranged between 9 and 20) in NOS2A proximal promoter pentanucleotide microsatellite.^31^ In Japanese asthma patients, Hirai et al (2018) suggested that alleles with <11 repeats of CCTTT contribute to poor asthma treatment outcomes when they found that carriers of alleles with <11 repeats were at a higher risk of asthma exacerbation. However, a 12‐repeat CCTTT polymorphism was found to be associated with high serum total IgE levels and serum NO levels.^15^

A single nucleotide polymorphism, the G894T, in the NOS3 gene has been described in asthmatic patients by at least five authors. In the present study, a meta‐analysis of four studies also could not find a significant association between G896T polymorphism and asthma. Holla et al (2002) studied atopic asthma in Czech population, and Gao et al (2000) studied in British population.^13^, ^16^ Both these authors found no significant association between NOS G894T polymorphism and asthma. van's Gravesande et al (2003) also did not find a significant association between G894T mutation and asthma. There was also no association between G894T mutation and either FEV1 or change in FEV1 post‐albuterol treatment in this study. However, T allele at this locus was associated with lower NO levels.^28^ Iordanidou et al (2012), who also did not find a significant association between G894T polymorphism and asthma, found that the G894T frequency was significantly higher in asthmatic children who responded to inhaled corticosteroids along with long‐lasting beta2‐agonists in comparison with non‐responders.^20^

Among the other polymorphisms, there was no significant association between asthma and a single nucleotide polymorphism in NOS3 gene, the −786 T/C, as observed by two studies in Czech and Greek Caucasian patients.^19^, ^20^ The −786 T/C polymorphism in NOS3 retards the transcription of NOS3 gene, which causes reduction in NO production up to 40%.^32^ Shakhanov et al (2007) reported that T allele of NOS3 −786C/T polymorphism increased the probability of bronchial asthma with comorbid essential hypertension 2.4 times in Russian asthma patients.^27^

Grasemann et al (2000) found a significantly decreased risk of asthma with NOS1 allele‐18 containing polymorphism in exon 29 but an increased risk with allele 17 at this locus in the Caucasian patients residing in the USA. They found no association between these mutations and IgE levels.^14^ Gao et al (2000) reported that in NOS1 microsatellite, homozygous 183‐bp alleles were significantly associated with asthma but not with atopy in British patients.^13^ Wechsler et al (2000), who studied a cohort of asthmatic patients consisting mainly of US white individuals, speculated that asthmatics harboring two alleles with 12 or more AAT repeats in NOS1 gene may have diminished exhaled NO levels.^29^ NO levels are found variable depending on the presence of single nucleotide polymorphisms in NOS genes.^33^

Yanamandra et al suggested that the variable tandem number of repeats in intron 4 of NOS3 appears to be a risk factor for asthma development in Caucasians or African Americans residing in the USA.^30^ Holla et al (2006) found that the asthma severity differed for different Ser608Leu exon 16 variants of NOS2 gene in Czech patients. Forty‐one percent patients with mild to moderate asthma had more active T allele of Ser608Leu; whereas, only 12% patients with intermittent form of asthma had this allele.^18^ Batra et al (2007) found a significant association of allele 3 with 15‐repeat GT in intron 4 of NOS2A gene with higher risk of asthma, asthma severity, and eosinophil percentage in peripheral blood in Indian asthma patients.^11^

Taken together, several lines of evidence suggest the role of polymorphisms in NOS genes to affect asthma etiology or prognosis. However, data are varying depending on the nature of variant, ethnicity, study and comparison design differences, and disease parameters. Moreover, a very few polymorphisms are studied in different ethnic groups or localities. Thus, replication of available data will refine the evidence regarding the role of various NOS gene polymorphisms in affecting the etiology and prognosis of asthma.

## AUTHOR CONTRIBUTIONS

Zeru Fan wrote the manuscript; Tao Liu and Wei Na collected and analyzed the data. All authors read and approved the final manuscript.

## CONFLICT OF INTEREST STATEMENT

None.

## ETHICS STATEMENT

N/A

## Data Availability

The datasets used and analyzed during the current study are available from the corresponding author on reasonable request.
